# Electronic health records model to improve the quality of patients’ healthcare

**DOI:** 10.4102/hsag.v29i0.2414

**Published:** 2024-09-17

**Authors:** Lovemore Motsi

**Affiliations:** 1 School of Computing, College of Science, Engineering and Technology, University of South Africa, Johannesburg, South Africa

**Keywords:** electronic health records, medical healthcare professionals, public hospitals, nurses, doctor

## Abstract

**Background:**

Electronic health records (EHR) has been acknowledged for bringing down healthcare costs and enhancing hospital service standards.

**Aim:**

The aim of this study was to develop an EHR model to lower patient treatment costs and enhance healthcare quality in South African public healthcare.

**Methods:**

In this study, a cross-sectional quantitative methodology was used. The research data for this study were provided by medical healthcare professionals, at Dr. George Mukhari Academic Hospital (DGMAH). This included doctors, nurses, pharmacists, radiologists, and radiographers who completed a semi-structured questionnaire.

**Results:**

The final model’s findings show that the use of EHR significantly improves information quality (IQ) and positively influences medical errors reduction (MER). Knowledge quality (KQ) has a positive significant impact on MER, whereas IQ has a considerable negative impact on MER. Furthermore, cost reduction in patient treatment (CRPT) has a positive significant influence on MER.

**Conclusion:**

Patients obtain better medical care when medical professionals have access to complete and accurate information. Medical errors can be reduced or even prevented with the use of EHRs, which can lead to better patient outcomes.

**Contribution:**

The quality of patient care at South African public hospitals and in other developing countries can be enhanced by using this framework as a guide to reduce treatment costs.

## Introduction

To address the unaffordable rising costs of healthcare, many governments in developed nations, including those in the United States (US), France, Germany, and the United Kingdom (UK), are promoting initiatives through regulations or monetary incentives to hasten the adoption of Electronic Health Records (EHRs) by primary care providers as well as hospitals (Walsham 2020). Electronic health records, a developing phenomenon, are the cornerstone of modern healthcare systems in the information age, and their use ‘may constitute a deviation from the standard of care’ (Atasoy et al. 2019). There has not been much research on EHR implementation in hospital settings, even though hospitals make up a significant amount of overall healthcare costs (van Poelgeest et al 2021).

Nevertheless, to guarantee uniform, comprehensive instructions, the term ‘EHR’ designates a collection of EHRs that contain information about medical histories, medications, physical examination results, physician reports, and staff notes (Eduhealthsystem 2022). The ability of departments within healthcare organisations to exchange patient medical data is one of the primary benefits of EHR, as it facilitates improved workflow (Woldemariam & Jimma 2023). Furthermore, the implementation of an EHR has the potential to accelerate the diagnostic and clinical decision-making processes by facilitating the instant access to all diagnostic information.

In addition, these advantages can be added to those previously mentioned, such as the capacity to avoid medication allergies and prescription duplication through simple access to patient medical records (Jabour 2020). However, EHR may provide certain challenges for patients, medical professionals, and healthcare organisations (Ngugi et al., 2021). Privacy issues, as well as the potential for less direct eye contact and conversation as doctors are entering data into the system while consulting with patients, are among people’ top anxieties with EHR (Ngugi et al. (2021); Zhang, Yu & Shen 2012). According to Woldemariam and Jimma (2023). EHRs have decreased the frequency of missing patient records by enabling doctors to access patients’ earlier diagnoses and treatments, hence enhancing the standard of care. Electronic health records improve medical records, lessen medical error rates, and cut healthcare costs in general (Bisrat, Minda, Assamnew & Abegaz 2021). The EHRs ensure proper data security and protection, enhance care efficacy, efficiency, and productivity in the healthcare sector. In addition, when medical personnel have access to their patients’ health information through EHRs, they can immediately analyse a patient’s test results, medical history, and other relevant clinic information (Bisrat et al. 2021).

In South Africa, most hospitals have relied on storing data manually using several classification schemes. (Msomi 2020). Hospitals have recently turned to employing EHRs systems for their everyday operations to improve service delivery. For the adoption and deployment of an EHR system to be successful, it is crucial to comprehend the factors influencing change management and how you may contribute to it. According to Jabour (2020), EHR systems are made to maintain the organisation of records while verifying their content, framework, and interactions with one another to promote accessibility and maintain value for hospital referrals. The difficulties South Africa’s public health system faces in maintaining records have been extensively studied by several authors (Erasmus & Van der Walt 2015; Katuu 2015; Luthuli 2017; Colicchio et al. 2019). As paper-based records are the only reliable source of information about patients’ medical histories, the state of public hospitals in the province of Limpopo is chaotic because of a lack of backup, safety and security measures, and disaster preparedness measures (Marutha 2019). Healthcare practitioners’ workload is negatively impacted, and patient health is directly impacted by the inability to retrieve medical records. Healthcare professionals are not permitted to treat patients who are experiencing problems, particularly if they are chronic and need therapy for several diagnoses with prescriptions for different medications, according to Luthuli (2017), Marutha (2018), Marutha and Ngoepe (2018), and other sources.

According to Luthuli and Kalusopa (2017) and Katuu and Van der Walt (2016), medical professionals working in healthcare facilities need constant access to medical information in order to deliver prompt patient treatment. An appropriate classification and security should always be in place for well-preserved records, and access should only be granted with the required authority or privileges. However, the entire range of challenges with managing change during the implementation process was not fully considered. Sulaiman and Magaireah (2014) investigated the factors that significantly influence the assumption of cloud-based consolidated e-health record EHR systems in healthcare facilities in Jordan using the Technology, Organisation, and Environment (TOE) scenario. The DeLone and McLean IS Success Model (D&M IS Success Model) (1992, 2003) served as the foundation for this investigation. To reduce patient treatment costs and improve healthcare quality this study set out to develop an EHR model for a public healthcare system in South Africa.

**FIGURE 1:**
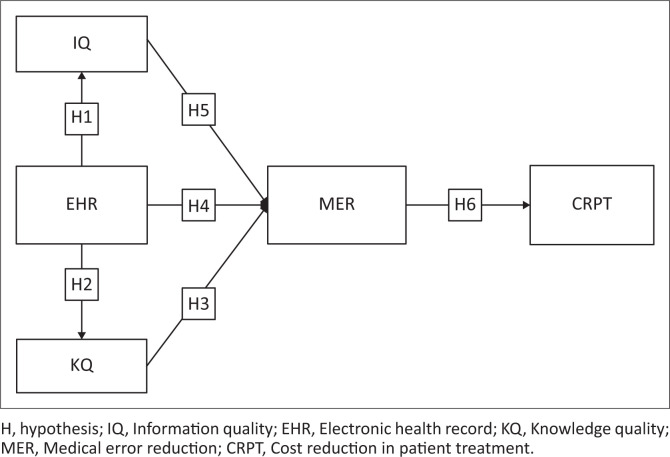
The conceptual framework.

The researcher developed a conceptual framework as shown in Figure 1 aimed at identifying factors that influence accurate disease diagnosis and treatment to enhance clinical treatment decisions made by doctors and other healthcare professionals. This will encourage preventative measures by giving patients a better understanding of their health, raise the speed and accuracy of identifying individuals at the highest risk of disease, and improve the efficiency of healthcare delivery, which in turn will cut costs. The conceptual model and research hypothesis are discussed in the following section.

### Theoretical framework

The fundamental theoretical underpinning for the research model employed in this study was the updated information systems success model developed by DeLone and McLean in 2003. One of the success constructs included in the model’s six aspects of success variables was net benefits, along with system quality, information quality (IQ), service quality, user satisfaction and other success constructs (DeLone & McLean 2003; Hassanzadeh, Kanaani & Elahi 2012; Holsapple & Lee 2006; Roca et al. 2006). In this study IQ was adopted, and changes were made to the construct. The system quality was adopted, and was modified to read EHR. In addition, the DeLone and McLean model was also expanded in this study with the addition of a new construct knowledge quality (KQ) that has not yet received empirical validation (De Zubielqui et al. 2019). The construct net benefit was updated to read cost reduction in patient treatment (CRPT) and another new construct, which was introduced to *DeLone and McLean* (*D&M*) information systems (IS) success model was medical error reduction (MER). The developed conceptual framework is shown in Figure 1.

The following research hypotheses (H1 and H2) as depicted in Figure 1 are directly influenced by EHR. H3: KQ has a direct impact on MER, according to the research framework. In this study EHR: H4 was hypothesised to have an influence on MER, while H5: IQ was predicted to have a positive significant impact on MER. Furthermore H6: MER was also hypothesised to have positive direct effect on the dependent variable CRPT. Using the structural equation modelling (SEM), the six proposed hypothesises in the conceptual framework were validated and tested. Table 1 shows SEM hypotheses results.

**TABLE 1:** Structural equation modelling results of hypothesis testing.

Relationship between the variables				Standardized coefficient	S.E	*p*-values	Result
EHR	→	H1	IQ	0.590	0.120	0.000	Accepted
EHR	→	H2	KQ	0.051	0.009	0.480	Rejected
KQ	→	H3	MER	0.604	0.198	0.000	Accepted
EHR	→	H4	MER	0.250	0.165	0.006	Accepted
IQ	→	H5	MER	0.164	0.173	0.038	Accepted
MER	→	H6	CRPT	0.170	0.187	0.049	Accepted

IQ, information quality; EHR, electronic health record; MER, medical error reduction; KQ, knowledge quality; CRPT, cost reduction in patient treatment; SE, standard error; H, hypothesis.

## Methods

### Participants, setting, and study design

#### Study design

To investigate, identify, and analyse the underlying factors that influence healthcare professionals’ decisions to adopt and use e-health technology applications in developing countries, a cross-sectional study design was used in this study, with reference to public hospitals in South Africa.

#### Setting

The setting for the study was Dr. George Mukhari Academic Hospital (DGMAH), an academic hospital in Ga-Rankuwa, of Gauteng province, Tshwane Region.

#### Population and sampling

Structural equation modelling was used in this investigation as it works well with high sample sizes. According to (Hair et al. 2019). the vast sample size enables consistent, repeatable results. According to Hair et al. (2022), 200 samples are the bare minimum for SEM. A value of 200 or above would ensure the validity of SEM. In this study, a sample size of 470 respondents was found adequate as suggested by Etikan, Alkassim and Abubakar (2016); (Forsberg & Rantala 2020). The healthcare professionals comprise a diverse range of professions and occupations that offer healthcare services. These include allied health professionals such as phlebotomists, medical laboratory scientists, dieticians, and social workers, as well as direct care practitioners like physicians, nurse practitioners, respiratory therapists, dentists, pharmacists, speech-language pathologists, physical therapists, occupational therapists, and behavioural and physical therapists (Teshome et al. 2019). This study population consisted of healthcare professionals (nurses, doctors, pharmacists, gynaecologists, urologists, radiologists, physiotherapists, dentists). Out of these 104 (38.1%) were men and 169 (61.9%) were women. This indicates that the sampled population’s thoughts were representative of both the research population’s genders.

In general, the gender distribution of the studied population appeared balanced and represented the national demographic traits of the population of healthcare professionals. A total of 34.7% of respondents, or the majority, were between the ages of 31 and 40. A total of 8.8% of respondents were between the ages of 41 and 50, 35.9% were between the ages of 25 years and 30 years, and 4.0% were older than 50. The majority of respondents fall into an informed age range, according to the results, and as a result, it is unlikely that they made well-informed decisions regarding the study.

The study’s medical healthcare participants have over 5 years of experience in public healthcare. Of them, 195 (29.2%) had worked in public service for 6 years–10 years, 156 (23.4%) for 11–15 years, 68 (10.2%) for more than 21 years, and 23 participants (3.4%) did not specify. This implies that participants with more than 5 years of experience ought to possess adequate understanding and proficiency to address the inquiries. Twenty-four respondents, or 24.9% of the sample, held master’s degrees, while the majority, 64.1%, had bachelor’s degrees. The percentage of healthcare professionals with a high school diploma was one (1), or 0.4%, while the percentage of responders in the other category was just three (3), or 4.0%. This indicates that the majority of respondents had a good education and could provide thoughtful, impartial feedback on the study. The distribution of medical specialists was based on functional area. A total of 179 (26.8%) of the participants were found to be from the outpatient department, with 140 (21.0%) coming from the intensive care unit, 94 (14.1%) from the X-ray department, 57 (8.5%) from the pharmacy department, and 37 (5.5%) from the radiology department. Nonetheless, 142 individuals (21.0%) did not name a specific hospital department, which is a significant percentage.

#### Sample selection

Elfil and Negida (2017) claim that convenience sampling is a non-probability sampling strategy that selects members of the target population who are in the location at a specific time. This helped to obtain access and time from medical healthcare professionals, including doctors, nurses, pharmacists, radiologists, and radiographers, during the coronavirus disease 2019 (COVID-19) pandemic, which was made extremely difficult by hospital restrictions aimed at reducing the spread of the virus convenience strategy was essential. Data were collected at DGMAH via self-administered surveys.

#### Data-collection tools

The survey method that was chosen for data collection was a self-administered questionnaire. Process of developing a questionnaire is intricate and requires careful thought (Grove & Gray 2022). In order to ensure that the questionnaire collects the most accurate data possible to meet the study’s aims, these factors need to be contextualised and linked with the research (Brace et al 2018). Therefore, it is the researcher’s duty to make sure the questionnaire is created in a way that would allow the respondents to carry out their duties in an appropriate manner (Sekaran & Bougie 2020). In addition, the suggested framework in Figure 1 served as the basis for developing the questionnaire. For this study, a two-part questionnaire was developed. The first section’s topic was demographic data about the respondents. The next section contained questions about the endogenous and exogenous latent variables. Exogenous constructs in the model are limited to independent variables utilised for predicting other constructs.

Endogenous constructs, on the other hand, behave as dependent variables that are predicted by a structural model (Hair et al. 2022). Information quality, EHR, KQ, MER, and CRPT are the independent variables in this study. A total of 49 questions were chosen from previous studies (DeLone & McLean 2003; Nguyen, Bellucci & Nguyen 2014). The responses were scored using a five-point Likert scale that ranged from ‘strongly disagree (1)’ to ‘strongly agree (5)’. To reduce patient treatment costs and improve healthcare quality, this study set out to develop an EHR model for a public healthcare system in South Africa.

#### Questionnaire validation through experts

In this study, expert opinions were used for pretesting the questionnaire. The questionnaire items were firstly, evaluated for validity by two academics from the School of Computing and a highly qualified colleague from the University of Mpumalanga, which had published numerous journal publications on e-health. Construct items were adjusted according to expert’s opinion. Initial testing of questionnaires often involves small samples, typically consisting of 20–40 individuals. According to Hair et al. (2019), small samples, however, might not be able to identify issues or accurately analyse the data collected on a small scale. For the pilot study, a default sample size of 80–100 individuals are generally advised (Hertzog 2008). Thus, prior to the researcher conducting the main study, the questionnaire was also tested through a pilot study with 20 medical healthcare experts at Tshwane District hospital to evaluate the general feasibility. In addition, the reliability of the constructs was tested, and all the constructs have a value of at least 0.70 for Cronbach’s alpha. The results show that there is appropriate internal consistency: KQ = 0.910, MER = 0.881, EHR = 0.871, and CRPT = 0.915. Information quality (IQ) = 0.862.

### Data analysis

The 300 completed surveys were then sorted, and the data were entered into the Statistical Package for the Social Science (SPSS) also known as IBM SPSS Statistics v20 and it was used to analyse data in this study. Each question was analysed using a coding system that summarised the responses into subjects. The SPSS software package was used to conduct the statistical analysis. Methods for multivariate analysis were used to examine the research data. Firstly, Cronbach’s alpha coefficient, whose value was required to be greater than 0.6 (Hair et al. 2019) and the item-total correlation, which was required to be greater than 0.3, were used to evaluate the scale reliability of the study model’s constructs (Afthanorhan et al. 2020).

To find latent constructs, data were reduced using exploratory factor analysis (EFA). The validity (both convergent and discriminant) and reliability (both convergent and discriminant) of these factors were assessed using confirmatory factor analysis (CFA). At the 5% level of significance, SEM was used to determine the effects of the factors on the intention to utilise digital banking services. When the Chi-square/*df* conditions were less than 3, the comparative fit index (CFI); incremental fit index (IFI); Tucker-Lewis Index (TLI) values were all greater than 0.9, and the RMSEA’s coefficient was less than 0.05, CFA, critical, and SEM analyses were reliable. (Hair et al. 2019; Hooper, Coughlan & Mullen 2008). Convergent validity was defined as a construct with all factor loadings of items larger than 0.5, and discriminant validity as a construct with the squared root of the variance greater than the correlation with other constructs (Hair et al. 2019).

### Ethical considerations

The study was carried out with ethical approval from the University of South Africa: University Research Ethics Committee (Reference No. [2019/CAES/075]) and consent from the Director of Clinical Services DGMAH. After obtaining approval from both the ethic committee as well as the permission from DGMAH the data-collection process was conducted in 2021 between the months of June and October. To maintain anonymity, no personal information about respondents was collected on the questionnaire. The fact that their participation was optional and that the information they provided would be kept private was made clear to the participants.

The participants’ demographics, construct item reliability measures, exploratory and CFAs, SEM, discussion of the research findings, conclusion, limitations, and future research directions will all be covered in the next section.

## Results

### Response rate and demographic characteristics of respondents

Primary data from 470 medical healthcare professionals at a public hospital in the province of Gauteng were gathered using a questionnaire survey method. A total of 300 useable questionnaires were produced by the survey, with a response rate of 63.8%. In this instance, women made up 61.9% of the respondents, with men making up the remaining 38.1%. A total of 35.9% of participants were between the ages of 25 and 30; 9.5% were younger than 25 and 37.4% of individuals were between the ages of 31 and 40. Only 19 (6.3%) of the participants had experience of less than 1 year, whereas 44.4% had an experience of 6–10 years, 34.3% had more than 10 years, 15.3% had less than 1 year. Nurses made up 75.0% of the respondents; doctors made up 4.0%; and pharmacists, radiologists, physiotherapists, and dentists made up 8%. A total of 1.0% of the respondents were gynaecologists, 0.3% were urologists, and 9.0% were from other professions. A bachelor’s degree was held by 64.1% of the respondents, a diploma by 68.9%, and a master’s degree by 6.2%. Compared to 0.4% who have a high school diploma and 0.4% who have a doctorate, 4.0% have a Bachelor of Medicine and Bachelor of Surgery (MBCHB). Table 2 displays the study sample’s demographic information.

**TABLE 2:** Demographic information of the sample.

Demographics	Category	Frequency	Percentage
Gender	Male	89	29.6
	Female	211	70.4
Age (years)	Less than 25	26	8.6
	25–30	98	32.6
	31–40	102	34.0
	41–50	57	19.0
	More than 50	17	5.6
Occupation	Medical doctor	16	5.3
	Pharmacist	12	4.0
	Radiology	10	3.3
	Physiotherapist	9	3.0
	Nurse	243	81.0
	Dentist	10	2.6
Work experience	Less than 1 year	19	0.6
	2–5 years	68	27.6
	6–10 years	147	49.0
	More than 10 years	76	25.3

### Construct item’s reliability measures

Instead of focusing on the dependability of a single variable, construct reliability (CR) assesses the internal consistency of a group of measurements. It assesses how strongly a group of measurements point to a shared latent component. Construct reliability has the benefit of being based on estimates of model parameters. To determine whether there were any correlations between the items and whether the constructs’ Cronbach’s alpha values might be raised, the items’ reliability was examined. As previously stated, a construct was considered credible if its Cronbach’s alpha was greater than 0.6 and its item-total correlation was greater than 0.3. Items having a lower than 0.3 item-total correlation coefficient would be removed from the scale and deemed unneeded. As a result, this item would not be considered later in this study. Table 3 displays the reliability measures for the construct item.

**TABLE 3:** Construct item’s reliability measures.

Constructs	Construct items	Cronbach’s alpha	Item-total correlation	Constructs removed
Electronic health records (EHR)	EHR1	0.627	8.075	EHR5, EHR6
	EHR2	0.786		
	EHR3	0.807		
	EHR4	0.675		
Medical error reduction (MER)	MER1	0.765	7.944	MER4, MER5, MER6
	MER2	0.786		
	MER3	0.548		
Information quality (IQ)	IQ3	0.829	7.007	IQ1, IQ2, IQ6
	IQ4	0.861		
	IQ5	0.783		
Knowledge quality (KQ)	KQ2	0.703	7.899	KQ1, KQ5, KQ6
	KQ3	0.788		
	KQ4	0.497		
Cost reduction in patient treatment (CRPT)	CRPT1	0.701	7.971	CRPT5, CRPT6
	CRPT2	0.726		
	CRPT3	0.735		
	CRPT4	0.534		

The construct items EHR5, EHR6, MER5, MER6, IQ1, IQ2, IQ6, KQ1, KQ5, and KQ6 were eliminated. They were eliminated because CRPT5 and CRPT6 had item-total correlation coefficients (0.238) that were less than 0.3. Following the deletion, the remaining constructs items were found suitable for study (Cronbach’s alpha > 0.6 and item-total correlation coefficients > 0.3).

### Exploratory factor analysis and confirmatory factor analysis

Confirmatory factor analysis was used to ensure that the data fit the model. The following outcomes of confirmatory component analysis were found: CFI = 0.922; TLI = 0.922; IFI = 0.923 better than 0.9; RMSEA = 0.059 less than 0.08; and Chi-square/*df* = 1.69, less than 3. It followed that the data, and the proposed model, were compatible. Reliability was evaluated using Cronbach’s alpha, CR, and average variance extracted (AVE). Convergent validity assesses the degree of correlation between two measures of the same concept. According to Hair, et al. (2019), high correlations demonstrate that the scale is assessing the concept it was intended to measure. The construct’s validity included both convergent and discriminant validity. Table 4 displays the outcomes of the convergent validity and Cronbach’s alpha tests for the 15-item CFA model of organisational components. Convergent validity could be seen because all the item factor loadings were higher than 0.5.

**TABLE 4:** Cronbach’s alpha, construct reliability and average variance extracted.

Constructs	Items	Factor loading	AVE	CR
Electronic health records (EHR)	EHR1	0.627	8.075	0.835
	EHR2	0.786	-	-
	EHR3	0.807	-	-
	EHR4	0.675	-	-
Medical error reduction (MER)	MER1	0.765	7.944	0.830
	MER2	0.786	-	-
	MER3	0.548	-	-
Information quality (IQ)	IQ3	0.829	7.007	0.833
	IQ4	0.861	-	-
	IQ5	0.783	-	-
Knowledge quality (KQ)	KQ2	0.703	7.993	0.812
	KQ3	0.788	-	-
	KQ4	0.497	-	-
Cost reduction in patient treatment (CRPT)	CRPT1	0.701	7.972	0.765
	CRPT2	0.726	-	-
	CRPT3	0.735	-	-
	CRPT4	0.534	-	-

IQ, Information quality; EHR, Electronic health record; KQ, Knowledge quality; MER, Medical error reduction; CRPT, Cost reduction in patient treatment; AVE, Average variance extracted; CE, Composite reliability.

According to Hair et al. (2018), all standardised factor loadings ought to be at least 0.5 and preferably 0.7. Construct had a composite reliability of 0.7 and an AVE above 50%. This proved that the construct-based scales that were used were adequate (Note 4 in Table). Table 5 shows that each construct’s square root of AVE was significantly higher than the correlation between the constructs, proving the discriminant validity of the scales.

**TABLE 5:** Discriminant validity.

Construct	IQ	EHR	MER	KQ	CRPT
IQ	**0.860**	-	-	-	-
EHR	0.607	**0.835**	-	-	-
MER	0.541	0.529	**0.847**	-	-
KQ	0.318	0.303	0.338	**0.823**	-
CRPT	0.647	0.737	0.561	0.313	**0.811**

Note: The values in bold represent the square root of the AVE scores.

IQ, information quality; EHR, electronic health record; MER, medical error reduction; KQ, knowledge quality; CRPT, cost reduction in patient treatment.

### Structural equation modelling

**FIGURE 2:**
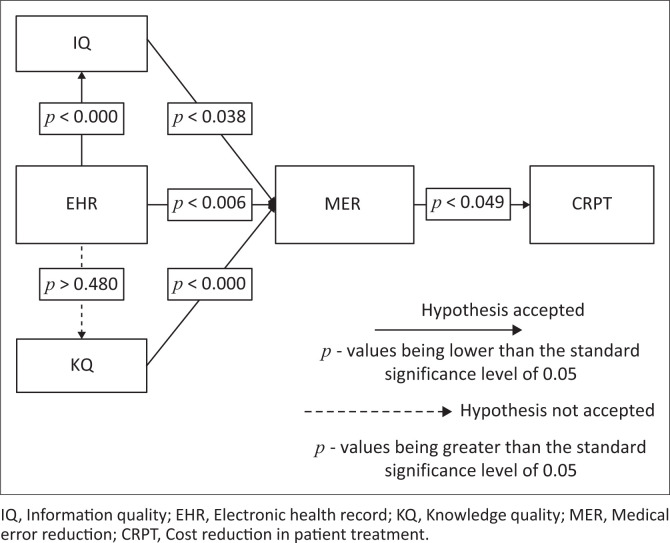
Model depicting accepted and unaccepted hypotheses.

The associations between a set of continuous latent variables and a set of observable variables are examined using CFA (Kueh et al 2017). Using a structural model, the relationship between the latent variables was investigated, according to Hair et al. (2019). The model was acceptable for the survey data, as shown by the analysis’s SEM results (Chi-square/*df* = 1.84 less than 3, CFI = 0.915; TLI = 0.90, IFI = 0.916 greater than 0.9, RMSEA = 0.065 less than 0.08). Structural analysis was used to investigate the effects of EHR on IQ (patient medical history), KQ (sharing of patient medical records among healthcare professionals), MER, and how these constructs will contribute to CRPT in South African public hospitals after testing the constructs’ validity and reliability. *P*-values of 0.05 were used to find the independent factors that substantially affect the dependent variable, CRPT, given that a significance level of 5% was established. Figure 2 displays the model as well as the accepted and rejected hypotheses.

The findings of structural analysis demonstrated that EHR had a positive significant impact on IQ, which in turn had a positive significant influence on the MER. In addition, it was found that EHR as expected significantly influenced MER and KQ had a positive significant impact on MER. However, EHR showed no direct impact on KQ. Furthermore, it was discovered that MER had a positive impact on CRPT. In other words, the findings rejected H2 and accepted H1, H3, H4, H5, and H6. Table 1 also shows SEM results of hypothesis testing results.

The results of the structural analysis showed that the quality of information in EHR had a positive significant impact on the MER, which in turn had a positive significant impact on IQ. In addition, it was discovered that KQ had a positive significant impact on MER and that EHR considerably influenced MER as was expected. Electronic health records however, did not indicate any direct significant influence on KQ. In addition, it was found that CRPT benefited from MER. In other words, the results supported H1 and rejected H2 while supporting H3, H4, H5, and H6. The SEM findings of the hypothesis testing are also displayed in Table 1.

## Discussion

In this study, the model was acceptable for the survey data, as shown by the analysis’s SEM results (Chi-square/*df* = 1.84 less than 3, CFI = 0.915; TLI = 0.90, IFI = 0.916 bigger than 0.9, RMSEA = 0.065 less than 0.08). In addition, the result of the final model evidently demonstrates EHR has a positive significant effect on IQ also associated with MER (*p* < 0.000; *p* < 0.006) respectively. Overall KQ has positive significant influence on MER *p* < 0.000 while IQ significantly impacts MER *p* < 0.038. Furthermore, MER has a positive significant influence on CRPT *p* < 0.049. No association between EHR and KQ was evident (*p* < 0.480). Therefore, while H2 was rejected, the following hypotheses were accepted: H1, H3, H4, H5, and H6.

The results of the hypotheses also demonstrated that the reduction of medical errors and the quality of the information play a significant role in lowering the cost of patient medical care. This study’s findings and those from past investigations agree. Kruse et al. (2017) assert that the introduction of EHR systems is intended to support clinicians’ use of evidence-based decision-making and streamline providers’ workflow through efficient patient care coordination (Wani & Malhotra 2018). Existing literature has emphasised the benefits of implementing EHR, such as improved patient safety measures, better patient outcomes, and cheaper costs (Kruse et al. 2017). In conclusion, the findings of these hypotheses are in line with other research that suggests the accuracy of the data may have a significant impact on the efficacy and safety of an EHR implementation.

In contrast to earlier studies utilising SEM (Albashrawi & Motiwalla 2020; Chang et al. 2020; Dey et al. 2020), which could only pinpoint linear correlations between the components, the methodological advancements made in the current investigation stand out. Using SEM as a statistical method, the researcher in this study was able to assess alternative hypotheses while also accounting for error. It contains a few observables and hidden independent, mediator, and dependent variables (Newman et al. 2010). Furthermore, the validation of the validity and reliability of the factors of an instrument and the testing of the causal relationships among the factors in an assumptive structural model allowed the researcher to develop a framework that can be used as a guide in lowering patient treatment costs and improving healthcare quality in South African public healthcare as well as in other developing countries.

### Limitations and directions for future research

Although empirical information from one public hospital was acquired, no comparison with other public hospitals in the Gauteng province was made. A methodological issue in the study is the use of self-reported scales to quantify the research components. As a result, the results can be somewhat constrained by the level of participant objectivity. Future research may create both subjective (self-reported) and objective assessments to completely account for each component. Finally, this study uncovered divergent viewpoints in the literature regarding the quality of knowledge, namely the sharing of patient medical records among healthcare professionals, which should lead to precise patient diagnosis and treatment. More empirical research is therefore needed to ascertain the nature of this relationship. In addition, a larger sample size and a greater geographic area may have been used for the investigation to increase the applicability of the findings.

## Conclusion

The findings of this study suggest that EHRs facilitate collection and analysis of healthcare data, enabling delivery of better patient care by healthcare providers. Clinicians can utilise this data to identify trends and patterns that will improve their understanding of patient needs and enable them to make more precise diagnoses. According to the study’s final model, EHRs can improve patient–provider communication by letting patients access their medical records and get in touch with their healthcare team.

According to the study’s findings, EHRs can improve patient data accuracy by reducing the likelihood of human mistake compared to paper-based alternatives. Electronic health records also make it simpler for healthcare personnel to monitor patient data, resulting in more accurate and recent records, if the study’s findings are put into practise in South African public hospitals. These results are consistent with those of Ratwani et al. (2018), who found that as more people relocate across the nation to other provinces, EHRs will enable access to patient medical histories for medical professionals at any time and from any location.

The results of the study show that EHRs can increase patient safety by reducing errors and enhancing the accuracy of patient data. Electronic health records can assist in lowering adverse medication events, which are required in developing nations like South Africa and other nations with low incomes such as Malawi, Uganda, and Zimbabwe in order to lower the cost of patient treatment. This can be achieved by leveraging EHRs to notify clinicians of potential drug interactions and allergies. The integration of EHRs may lead to better patient outcomes by enhancing the accuracy of patient data, enabling better care coordination, and providing more individualised medical care.
